# Mr. Marson, in Answer to Mr. Ring

**Published:** 1807-02

**Authors:** W. Marson

**Affiliations:** Worksop, Notts


### *To the Editors of the Medical and Physical Journal.*

GENTLEMEN,

IN the last Number of the Medical Journal, I observe Mr. Ring attributes the frequent failure of Vaccination taking place to the fault of the matter, or the operation being imperfectly performed; I gave you my opinion a few months back, that it was owing to the non-absorption of the matter; and the purport of this letter, is to draw the public attention to that particular.

Mr. Ring speaks of respectable practitioners, not being conversant with the practice of Inoculation; I am persuaded if the matter is good, that no particular address is required in the operation; though I agree with Mr. Ring, that if the smallest quantity of matter is inserted, that is, absorbed, the inoculation will proceed; yet I cannot but conceive that this small quantity of matter in the twenty-nine times mentioned by Dr. Walker, must have adventitiously been successful, if it had depended on the operation, or the (changes of) matter employed. Mr. Ring will pardon me in maintaining, that it was owing to the inactivity, or call it what you will, of the absorbents.

Notwithstanding Mr. Ring has thought differently from me me, the case he mentions, (page 323,) leans very much to my proposal of medicine and regimen, for if the constitution of the patient had been healthy, the mere cutting up a vaccine vesicle, or irritation of the arm, would not have produced the symptoms he speaks of. I trust it will be allowed, there is such a thing as a bad habit; and although there are constitutions, where the inoculation may do well without regimen and medicine, yet it would seem necessary to be aware of, and guard against cases of the nature he describes; and in so doing, we have little to fear from the irritation of the arm, or the cutting up a vaccine vesicle. Dr. Willan, who though no inoculator, would I believe (from your review of his Publication) support me in this; he says, "there may be also a few, in which the inoculation excites a new mode of action, terminating in erysipelas or phagedenic ulcer, or other morbid appearances, not necessarily connected with the specific disease;" implying that it would be well to attend to the constitution previous to, or at the time, of the inoculation.

Much I observe, is said about tests; what I depend upon, if I am certain the matter is genuine, is the pulsation of the inoculated part; when I perceive this, I am certain it will proceed, and I think very little of the breakage of the vesicle.

I am, &c.

W. MARSON.

*Worksop, Notts. November* 11, 1806.

